# A Clinical Encounter With Pickwickian Syndrome

**DOI:** 10.7759/cureus.28778

**Published:** 2022-09-04

**Authors:** Puja Upadhyay, Ulhas S Jadhav, Gaurang M Aurangabadkar, Ajay V Lanjewar, Pankaj Wagh, Babaji Ghewade, Juhi Kadukar

**Affiliations:** 1 Respiratory Medicine, Jawaharlal Nehru Medical College, Datta Meghe Institute of Medical Sciences, Wardha, IND; 2 Respiratory Medicine, Datta Meghe Medical College, Datta Meghe Institute of Medical Sciences, Wardha, IND

**Keywords:** obesity, obstructive sleep apnea (osa), non-invasive positive pressure ventilation, obesity hypoventilation syndrome, pickwickian syndrome

## Abstract

The clinical syndrome described in the literature as "Pickwickian syndrome" is characterized by a combination of sleep-disordered breathing, obesity, and daytime hypercapnia; the condition is also known as obesity hypoventilation syndrome (OHS). This syndrome is a diagnosis of exclusion after every other possible etiology is ruled out. Patients can present both with an exacerbation of or a chronic state of progressive dyspnea. In this report, we describe the case of a 62-year-old morbidly obese female with a BMI of 42 Kg/m^2^, who presented with progressively worsening breathlessness. An arterial blood gas (ABG) analysis revealed severe hypoxia with hypercarbia. A sleep study [polysomnography (PSG)] of the patient was performed, which revealed an apnea-hypopnea index (AHI) of 58.2, and the patient was diagnosed as having OHS after all other possible cardiorespiratory etiologies were ruled out. The patient was promptly managed with non-invasive ventilatory (NIV) support along with supportive management and was prescribed overnight NIV and subsequently discharged in stable condition.

## Introduction

The name "Pickwickian syndrome" was coined after a fictional obese character named Joe in Charles Dickens's novel the “Pickwick Papers” [1]. There has been an inexorable rise in the number of cases of obesity worldwide in the past four to five years. India is undergoing a rapid epidemiological transition, from an underweight to an overweight/obese population. It can be attributed to the rapid lifestyle changes associated with high caloric intake and reduced physical activity, putting Indian people at a high risk of obesity. Obesity is associated with innumerable comorbidities including hypertension, diabetes mellitus, and cardiac complications [2]. Obesity hypoventilation syndrome (OHS), the prevalence of which has been shown to rise in direct proportion to the prevalence of obesity, is considered an important sequela of severe obesity. Patients with OHS experience a wide variety of difficulties ranging from congestive cardiac failure, metabolic syndrome, and obstructive sleep apnea (OSA). We report the case of a 62-year-old female who presented with severe dyspnea and, after polysomnography (PSG), was diagnosed with OHS and managed with non-invasive positive pressure ventilation (NIPPV) support.

## Case presentation

A 62-year-old female presented to the ED with complaints of breathlessness for six months, which had been initially mild but progressed to severe dyspnea in the last 15 days prior to the admission. The patient also had complaints of a productive cough and intermittent low-grade fever with chills for 10 days. She had consulted at a local hospital for the same complaints and received symptomatic management with inhaled bronchodilators and oxygen support, but did not show any clinical improvement and was referred to our tertiary care center in Wardha, India for further management.

Clinical examination and routine investigations

**Figure 1:**
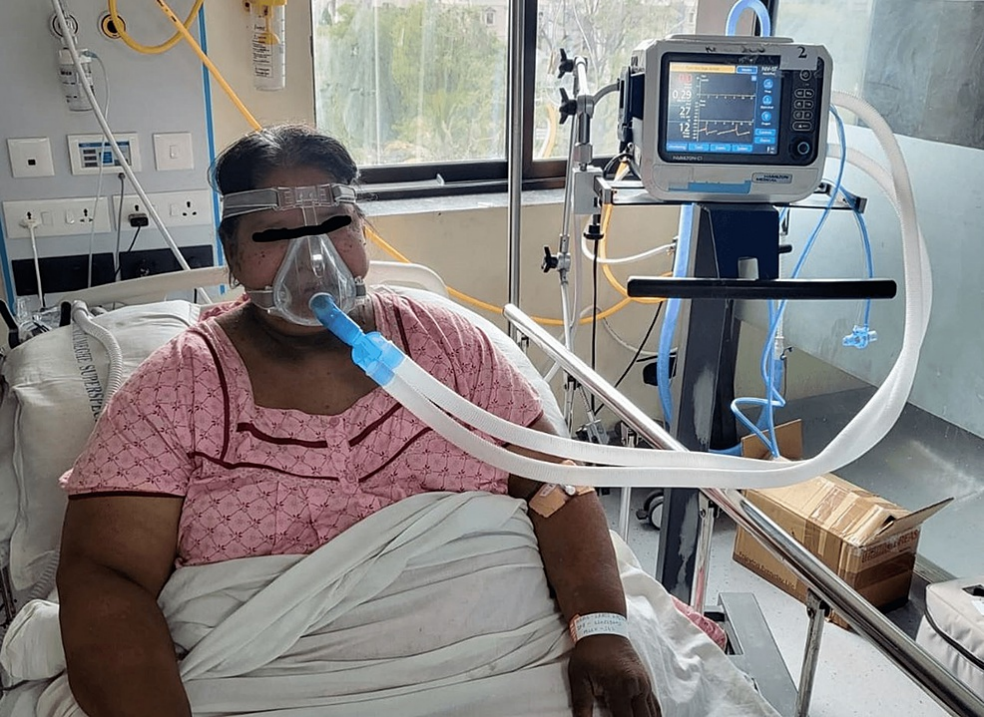
Clinical photograph of the patient in the ICU on non-invasive positive pressure ventilation (NIPPV) support

On general examination, the patient was found to have severe obesity with a BMI of 42 Kg/m^2^. The vital parameters of the patient were as follows - pulse rate: 98 beats/minute, blood pressure: 150/90 mmHg, respiratory rate: 28 breaths/minute, and oxygen saturation: 84% on room air on pulse oximetry. She also had bilateral pitting edema present on the lower limbs and a raised jugular venous pressure (JVP). No other obvious abnormalities were found on the rest of the clinical examination. The clinical image of the patient on admission to the ICU is shown in Figure 1.

The patient underwent arterial blood gas (ABG) analysis, which revealed respiratory acidosis along with hypoxia and hypercapnia; other routine blood investigations showed no abnormalities apart from elevated blood d-dimer levels. The results of significant blood investigations are summarized in Table 1.

**Table 1:** Routine blood investigations of the patient on admission to the ICU

Arterial blood gas analysis (ABG)	Patient values	Normal range
Potential of hydrogen (pH)	7.26	7.35–7.45
Partial pressure of carbon dioxide (pCO_2_)	70 mmHg	35–45 mmHg
Partial pressure of oxygen (pO_2_)	58 mmHg	80–100 mmHg
D-dimer levels	980 ng/ml	Less than 500 ng/ml

**Figure 2:**
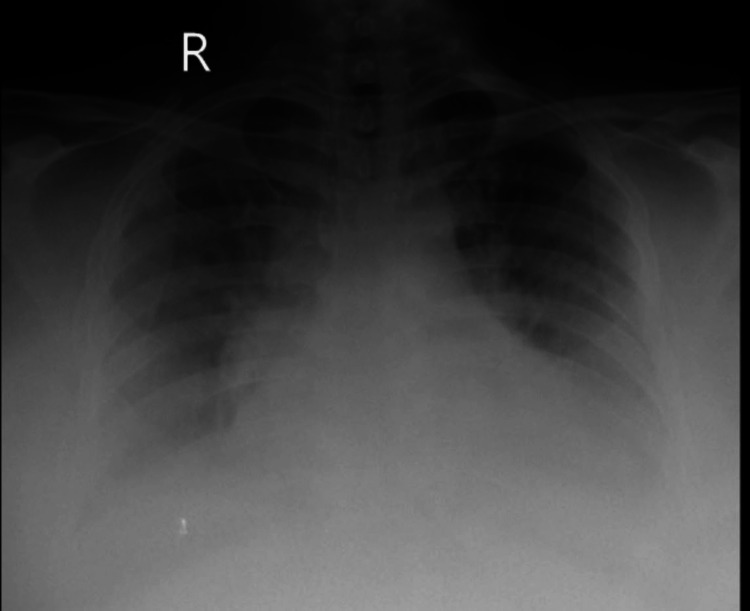
Chest X-ray anteroposterior (AP) view suggestive of bilateral lower-zone haziness

The patient was put on NIPPV support, but a repeat ABG analysis showed no improvements in her oxygenation status. She was intubated and put on mechanical ventilatory support in volume control (VC) mode. She was gradually weaned off mechanical ventilation and, following extubation after five days, was again put on NIPPV support. A detailed history of the patient did not reveal any major risk factors for chronic obstructive pulmonary disease (COPD). Further diagnostic imaging tests such as a chest X-ray and two-dimensional echocardiography (2D-echo) were done to rule out any comorbid cardiorespiratory conditions. The chest X-ray of the patient revealed bilateral mid-zone and lower-zone haziness, which may be seen in bilateral lower-lobe pneumonia or pleural effusion, as shown in Figure 2.

Ultrasonography (USG) of the thorax was done, which revealed no evidence of underlying pleural effusion. 2D-echo of the patient revealed a mildly dilated right atrium and right ventricle with a left ventricular ejection fraction (LVEF) of 55%. After the clinical stabilization of the patient, we conducted a PSG of the patient to investigate further causes of her respiratory failure, especially given her history of daytime sleepiness and excessive snoring. PSG revealed that the patient had an apnea-hypopnea index (AHI) of 58.2, which was classified as severe OSA as per a recent classification of OSA severity [3]. A summary of the PSG findings of the patient is given in Table 2.

**Table 2:** Polysomnography (PSG) findings of the patient showing severe obstructive sleep apnea (OSA) with an apnea-hypopnea index (AHI) of 58.2 RDI: respiratory disturbance index; REM: rapid eye movement sleep; NREM: non-rapid eye movement sleep

Respiratory disturbance index including respiratory effort-related arousals (RERA) and total sleep time (TST)			
	REM (episodes/hour)	NREM (episodes/hour)	TST (episodes/hour)
RDI	30	58.6	58.2
Apnea-hypopnea index (AHI) excluding central apneas			
	REM (episodes/hour)	NREM (episodes/hour)	TST (episodes/hour)
AHI	30	58.6	58.2
Hypopnea summary			
	Total events	With drops in heart rate	With drops in oxygen saturation
Total number	108	92	94
Max length (sec)	57.5	57	57.5
Central apnea summary			
	Total events	With drops in heart rate	With drops in oxygen saturation
Total number	187	149	145
Max length (sec)	50	50	50
Apneas preceded by sighing	8	7	6

**Figure 3:**
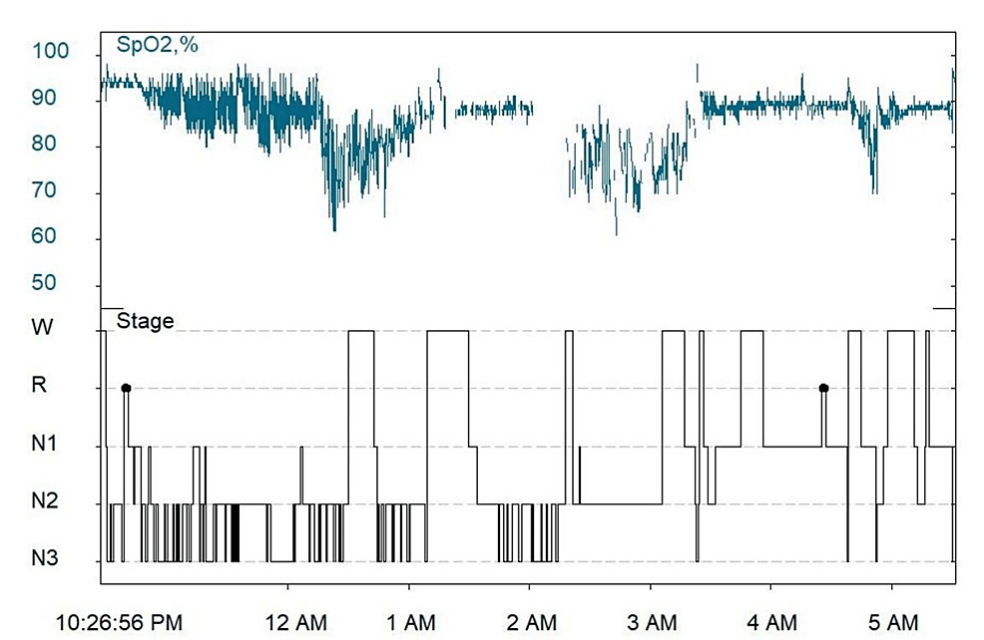
Hypnogram showing oxygen saturation variability in different stages of sleep

The hypnogram of the patient, showing multiple episodes of apnea and hypopnea and oxygen desaturation in various sleep stages, is shown in Figure 3.

After extensive investigations, we diagnosed the patient with OHS based on persistent daytime hypercapnia, OSA diagnosed on a PSG, and clinical findings of severe obesity. Overnight NIPPV support along with intermittent oxygen support during the daytime was continued. She was discharged after 15 days with stable vitals, with a prescription for overnight NIPPV support and a specialist consultation with a bariatric surgeon to consider possible weight-reduction interventions/surgeries.

## Discussion

After ruling out other pathologies that could lead to alveolar hypoventilation, OHS is characterized as the presence of obesity (BMI of 30 kg/m^2^), and daytime hypercapnia [arterial carbon dioxide tension (PaCo_2_) of more than 45 mmHg], with sleep-disordered breathing [2]. An upper airway obstruction, a deficiency in ventilatory drive, and obesity are the leading causes of OHS. Patients with OHS who are obese are forced to breathe at low functional residual capacity (FRC) with reduced diaphragm activity [3]. Indian patients have an OHS prevalence that is comparable to that of Caucasians, despite having a reduced BMI and spirometric findings [4]. All individuals with centrally distributed morbid obesity should get a thorough OHS evaluation. To confirm daytime hypercarbia and hypoxia, it is important to check for pulse oximetry data indicating awake hypoxemia and to conduct ABG analysis. To look into respiratory disorders related to sleep, a PSG should be carried out.

The patient in this report presented with severe hypoxia and hypercarbia needing mechanical intubation initially. This shows that obesity hypoventilation can present as acute illness or exacerbation where patients are more hypoxemic and more hypercapnic than usual. Proper management at such times becomes crucial. Therapeutic errors, especially at the time of hospitalization for respiratory or cardiovascular decompensation, should be avoided. Subjects with OHS may develop acute hypercapnia in response to the administration of excessive supplemental oxygen and excessive diuresis for peripheral edema using a loop diuretic such as furosemide exacerbates metabolic alkalosis, thereby leading to the worsening of daytime hypoventilation and hypoxemia [5]. Post-discharge management is essential for OHS patients. The most important component of managing OHS is weight loss [6]; yet, it is sometimes challenging to achieve and maintain weight loss with medical care. Bariatric surgery helps OHS patients lose weight more effectively and sustain it for longer periods of time. Recent research has revealed that using NIV to treat chronic respiratory failure is linked to weight loss and a reduction in sedentary time. In addition to NIV, a multimodal rehabilitation program can benefit OHS patients by enhancing weight loss and improving their ability to exercise [7].

## Conclusions

OHS is not very frequently diagnosed in general practice and hence its prevalence is underestimated. A patient presenting with hypoxia and hypercarbia with central obesity should be carefully evaluated for OHS and a proper plan of management should be implemented to avoid any detrimental consequences. NIPPV should be instituted at the earliest along with effective weight reduction strategies to improve the prognosis of the patient. A PSG should also be done in such patients to rule out sleep-disordered breathing. Obesity is a health condition affecting almost all body systems, and this case report highlights its detrimental effects on respiratory and sleep physiology, which, in this case, led to hypercapnic respiratory failure.
